# Route-Planning Method for Plant Protection Rotor Drones in Convex Polygon Regions

**DOI:** 10.3390/s21062221

**Published:** 2021-03-22

**Authors:** Shaoxing Hu, Tianliang Xu, Bingke Wang

**Affiliations:** School of Mechanical Engineering and Automation, Beihang University, Beijing 100191, China; daylight@buaa.edu.cn (T.X.); 1920wangbk@buaa.edu.cn (B.W.)

**Keywords:** route-planning, initial heading angle, coordinate conversion, subregion, route distance, number of turns, pesticide waste rate

## Abstract

Aiming at the problem of low operating efficiency due to the poor endurance of plant protection rotor drones and the small volume of pesticide carried, this paper proposes a route-planning algorithm for convex polygon regions based on the initial heading angle. First, a series of coordinate conversion methods ranging from the Earth coordinate system to the local plane coordinate system are studied. Second, in the local plane coordinate system, a route generation method based on subregion is proposed; therefore, multiple routes can be generated with different initial heading angles. Lastly, the optimal route and the best initial heading angle can be obtained after the comparison according to the three evaluation criteria: number of turns, route distance, and pesticide waste rate. The simulation results show that, compared with the common grid method, the route generation method based on subregion reduces the route distance and pesticide waste rate by 2.27% and 13.75%, respectively. Furthermore, it also shows that, compared with the route generated by the initial heading angle of 0°, the optimal route reduces the number of turns, route distance, and pesticide waste rate by 60%, 17.65%, and 38.18%, respectively. The route was optimized in three aspects and reached the best overall result using this method, which in turn proved its feasibility.

## 1. Introduction

Applying drones in plant protection owns the characteristics of uniform pesticide spraying, high efficiency, low cost, and high security; as such, drones have been widely used in the agriculture field [1,2,3,4]. Moreover, due to the large-scale circulation of land in China, especially in recent years, the demand for drone application has increased rapidly [5,6]. Under such a background, the key problem of agriculture spraying has gradually changed from whether the crops get sprayed to how the crops get well sprayed. To make sure that the crops get well sprayed, it is not only necessary to ensure the uniformity of the pesticide in the operation region, but also the accuracy of the spraying operation [7,8]. Currently, China mainly uses rotor drones for plant protection because they can ensure the uniformity of the pesticide on each crop in the operation region to a certain extent [9]. However, they have poor endurance and small pesticide capacity, thus reducing the efficiency of operation [10]. In order to maximize the energy utilization and the pesticide utilization of drones, it is urgent to develop a route-planning method to ensure that the crops get well sprayed while also improving the efficiency of operation.

The problem of route-planning for plant protection is a type of complete coverage problem (CCP), which is developed to obtain the shortest route or the lowest-cost route to cover all places except obstacles within a given region [11]. Choset [12] put forward that CCPs can be divided into two cases: online and offline. The difference between online CCP and offline CCP is whether the environmental information is known by developers in advance. When solving online CCP, developers often have little or partial information about the environment and need to obtain the information by calculation with the messages of real-time sensors [13]. While figuring out offline CCP, developers always know the environmental information, including the shape of the operation region, the area of the operation region, and the obstacles in the operation region [14]. The aim in this paper was to solve an offline CCP and obtain the lowest-cost route.

When it comes to offline CCP problems, the type of operation region given according to offline information can vary, such as single convex polygon region [15], single irregular region [16], single region with obstacles [17], and a region consisting of multiple regions [18]. However, the problem of route-planning in a complex region including an irregular region, region with obstacles, and combination of multiple regions can be decomposed into multiple problems of complete coverage in a convex polygon region and a problem of connecting multiple convex polygon regions. The authors of [19,20] proposed decomposition methods to simplify route-planning problems in complex regions, whereas the authors of [21,22] determined how to generate the optimal connection route between regions. By optimizing the route in a convex polygon region, the route in a complex region can also be optimized. What calls for special attention is that using the cooperative operation of multiple vehicles [23] can also solve the problem of route-planning in a complex region. However, each vehicle gets its assigned region, usually a convex polygon region, in the cooperative operation of multiple vehicles [24,25,26], which means that it is also useful to improve its efficiency by optimizing the route-planning problem in a convex polygon region. Accordingly, this paper focuses on obtaining the optimal route in a convex polygon region.

Moreover, when obtaining the lowest-cost route in an operation region, the evaluation criteria can be different [25]. Existing studies on coverage route-planning typically analyzed different routes according to one or several evaluation criteria, such as minimum energy [27,28], minimum flight time or flight distance [29,30], maximum coverage rate [31,32], and minimum number of turns [24,33]. In order to minimize energy consumption of the route, Di Franco [34] proposed an energy-aware path-planning algorithm based on an energy model derived from real measurements, and this algorithm also satisfied a set of other requirements, such as coverage and resolution. Vasquez-Gomez [35] put forward an optimal route-planning algorithm with the purpose of getting the minimum number of flight lines, and this paper took into account the starting and ending points. With the aim of optimizing the battery consumption of an unmanned aerial vehicle (UAV), Torres Marina [36] presented a route-planning method to minimize the number of turns and the total flight distance. Xu Bo took the total UAV flight distance and excess coverage rate as the evaluation criteria and proposed an optimal route-planning method based on the heading angle. 

However, the above research works mainly focused on reducing the energy consumption of a UAV or drone, which is not enough for practical operations of plant protection, where how to reduce the pesticide consumption is equally important. Furthermore, the uniformity of pesticide in the operation region should be considered when carrying out a plant protection task. In this paper, our aim was to ensure the uniformity of pesticide in the operation region and to achieve the route with minimal energy and pesticide consumption through an evaluation of the route distance, number of turns, and pesticide waste rate. In order to ensure the uniformity of the pesticide, this paper proposes a new route generation method based on subregion, and this method also makes sure that the energy and pesticide consumption of the generated routes is optimal, which allows analyzing the optimal route among different routes. To obtain different routes for comparison and analysis, this paper combines the new route generation method with different initial heading angles. To obtain the optimal route of plant protection operation, this paper quantitatively analyzes different routes with three evaluation criteria (route distance, number of turns, and pesticide waste rate) by assigning each criterion a weight.

**Figure 1:**
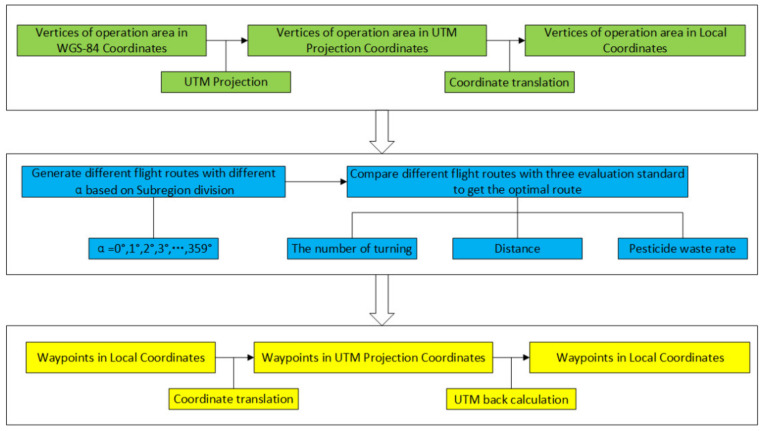
Overall flow chart of the route-planning method proposed in this paper.

This paper is organized as follows: first, the offline information is inputted, including the vertices of the convex polygon region and the takeoff and landing points. The inputted information is then converted from the Earth coordinate system to the plane coordinate system. Second, a route generation method based on subregion in the plane coordinate system is introduced in detail, and different routes with different initial heading angles are generated via the method. Then, the optimal route and the best initial heading angle are obtained after quantitatively analyzing different routes according to three evaluation criteria: route distance, number of turns, and pesticide waste rate. Furthermore, the optimal route is outputted in the Earth coordinate system through coordinate conversion. Lastly, the feasibility of this route-planning method is verified through simulation. The flow chart of the integrated method in this paper is given in Figure 1.

## 2. Materials and Methods

### 2.1. Coordinate Conversion of Input Operation Region and Takeoff Point

In order to plan the route in the convex polygon operation region, the operation region should be delimited, and the takeoff point should be established, assuming that the takeoff point and the landing point are the same point. The convex polygon operation region can be uniquely represented according to the coordinates of the given vertices of the convex polygon and their serial number. To determine the position of the operation region on the Earth to facilitate drone navigation, the WGS-84 coordinate system was adopted when inputting the operation region vertices and takeoff point. Because the WGS-84 coordinate system is a spherical coordinate system and route-planning can only be carried out using a plane coordinate system, it was necessary to convert the operation region vertices and takeoff point from the WGS-84 coordinate system to a plane coordinate system convenient for route-planning calculation. The coordinate conversion process can be divided into two processes: plane projection and plane coordinate system localization.

#### 2.1.1. Plane Projection

To ensure that the angle of the operation region is not deformed and the length and area of the operation region are not deformed after the coordinate system is converted from spherical to planar, the Universal Transverse Mercator (UTM) projection method was used and the obtained plane coordinate system was called the UTM projection plane coordinate system.

When using the UTM projection to project a point on the sphere into plane, the basic formula [37,38] is as follows:

(1) $$\left\{\begin{array}{l} x=FN+K_{\varphi }\left[\begin{array}{l} S_{\varphi }+\frac{\lambda^{2}N}{2}\sin\varphi \cos\varphi +\\ \frac{\lambda^{4}N}{24}\sin\varphi \cos^{3}\varphi (5-\tan^{2}\varphi +9{e^{\prime }}^{2}\cos^{2}\varphi +4{e^{\prime }}^{4}\cos^{4}\varphi )+\\ \frac{\lambda^{6}N}{720}\sin\varphi \cos^{5}\varphi (61-58\tan^{2}\varphi +\tan^{4}\varphi +270{e^{\prime }}^{2}\cos^{2}\varphi -330{e^{\prime }}^{2}) \end{array}\right]\\ y=FE+K_{\varphi }\left[\begin{array}{l} \lambda N\cos\varphi +\frac{\lambda^{3}N}{6}\cos^{3}\varphi (1-\tan^{2}\varphi +{e^{\prime }}^{2}\cos^{2}\varphi )+\\ \frac{\lambda^{5}N}{120}\cos^{5}\varphi (5-18\tan^{2}\varphi +\tan^{4}\varphi +14{e^{\prime }}^{2}\cos^{2}\varphi -58{e^{\prime }}^{2}) \end{array}\right] \end{array}\right.,$$

where x and y are the *X*-coordinate and the *Y*-coordinate of the point converted to the UTM projection plane coordinate system (the positive direction of the *X*-coordinate and the *Y*-coordinate corresponds to the geographical direction of east and north), FN is the offset of north, which is generally equal to 0 in the northern hemisphere and equal to 10,000,000m in the southern hemisphere, FE is the offset of east, which is generally equal to 500,000m, $K_{\varphi }$ is the UTM projection proportion coefficient of the central meridian, which is generally equal to 0.9996, $S_{\varphi }$ is the length from the central meridian intercepted by the parallel circle of the point to the equator, λ is the difference value between the longitude of the point and the longitude of the central meridian, φ is the latitude of the point, N is the curvature radius of the prime vertical at the point, and $e^{\prime }$ is the second eccentricity of the Earth.

Assuming the input vertices of the convex polygon operation region are $P_{1},\;P_{2},\;\ldots ,\;P_{m}$ in turns, m is the number of vertices of the convex polygon, and the coordinates of the *i*-th vertex $P_{i}\left(i=1,2,\ldots ,m\right)$ are $\left(\lambda_{i},\;\varphi_{i}\right)$, where $\lambda_{i}$ is the longitude of the vertex $P_{i}$, and $\varphi_{i}$ is the latitude of the vertex $P_{i}$; the input takeoff point is O, and the coordinates in the WGS-84 coordinate system are $\left(\lambda_{O},\;\varphi_{O}\right)$. By using Equation (1) to convert the coordinates of the vertices $P_{1},\;P_{2},\;\ldots ,\;P_{m}$ in the WGS-84 coordinate system, the coordinates $\left(x_{1}^{UTM},\;y_{1}^{UTM}\right),\;\left(x_{2}^{UTM},\;y_{2}^{UTM}\right)\;\ldots \;\left(x_{m}^{UTM},\;y_{m}^{UTM}\right)$ of vertices in the UTM projection plane coordinate system can be obtained. Similarly, the coordinates $\left(x_{O}^{UTM},\;y_{O}^{UTM}\right)$ of the takeoff point in the UTM projection plane coordinate system can also be obtained.

#### 2.1.2. Plane Coordinate System Localization

The coordinates of the vertices $\left(x_{1}^{UTM},\;y_{1}^{UTM}\right),\;\left(x_{2}^{UTM},\;y_{2}^{UTM}\right)\;\ldots \;\left(x_{m}^{UTM},\;y_{m}^{UTM}\right)$ and the coordinates of the takeoff point $\left(x_{O}^{UTM},\;y_{O}^{UTM}\right)$ obtained using the UTM projection are numerically large, which increases the amount of calculation. In order to reduce the amount of calculation caused by the large value, a coordinate system translation method was applied, and the UTM plane projection coordinate system was further converted into the local plane coordinate system with a smaller value of vertex coordinates. In the local plane coordinate system, the distance from each vertex of operation region to the origin should be smaller. Considering that, in the actual plant protection operation, the takeoff point should be close to the operation region, the UTM plane projection coordinate system can be translated to the position where the origin coincides with the takeoff point, whereby the obtained new coordinate system was called the local plane coordinate system. The formula of the localization procedure is as follows:

(2) $$\left\{\begin{array}{l} x_{i}=x_{i}^{UTM}-x_{o}^{UTM}\\ y_{i}=y_{i}^{UTM}-x_{o}^{UTM} \end{array}\right.\left(i=1,2,\ldots ,m\right),$$

where $x_{i}$ and $y_{i}$ are the *X*-coordinate value and the *Y*-coordinate value of the vertex of the operation region in the local plane coordinate system, corresponding to the geographical direction of east and north, respectively.

### 2.2. Route-Planning Method Based on Initial Heading Angle

After converting the vertices of the operation region and the takeoff point to the local plane coordinate system, route-planning can be carried out. The goal of route-planning is to get the optimal route and improve operation efficiency. In order to achieve this goal, different routes need to be generated, which are then compared according to the evaluation criteria to obtain the optimal route.

#### 2.2.1. Route Generation Method Based on Subregion

**Figure 2:**
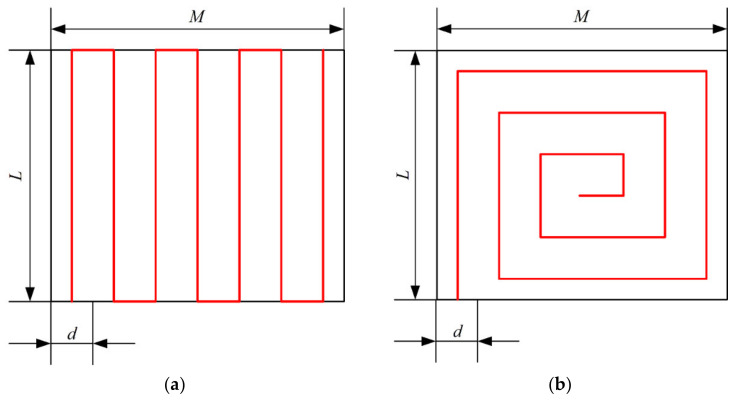
Two methods of covering the operation region: (**a**) reciprocating traversing method; (**b**) square spiral method. The black rectangle is the operation region and the red lines represent the route; parameter *d* denotes the spraying swath width.

Before generating the route, it is necessary to determine the way to cover the operation region. The two main methods are the reciprocating traversing method [39,40] and the square spiral method [26,41]. These two methods are shown in Figure 2. When comparing the routes using these two methods, the number of turns is the same, while the route using the reciprocating traversing method has a slightly longer route distance. However, the coverage using the reciprocating traversing method is more uniform during the spraying operation, with no repeated coverage and missing coverage [22]. To ensure the uniformity of pesticide coverage in the operation region, the reciprocating traversing method was selected as the way to cover the operation region. With the purpose of generating an operation route similar to Figure 2a and preventing a reduction in operation efficiency caused by route generation as much as possible, this paper proposes a route generation method based on subregion.

To simplify the problem, given the fact that most plant protection quadrotors in the market install nozzles under the four propellers, the following assumptions were made:

1. Drones are regarded as particles while flying.
2. When a drone flies to a certain point, the pesticide covering the shape are square in nature, where the length of one side is the spraying swath width.
3. In the square described in assumption 2, the pesticide sprayed at each point is uniform.
4. Drones only spray pesticide when they fly across the operation region.

On the basis of the above assumptions, the operation region can be divided into subregions along the *Y*-coordinate using the spraying swath width as the interval.

In the local plane coordinate system, the bottommost point D and the topmost point U of the *Y*-coordinate of the operation region, whose *Y*-coordinate values are set as $y_{\min}$ and $y_{\max}$, respectively, can be found. Then, the number of divisible subregions of the operation region can be calculated as follows:

(3) $$n=ceil(\frac{y_{\max}-y_{\min}}{d}),$$

where function ceil represents rounding up, and d is the spraying swath width.

The equation of the *k*-th subregion border line is as follows:

(4) $$y=y_{\min}+(k-1)d,k=1,2,\ldots ,n,n+1.$$

According to Equation (4), the range expression of the *j*-th subregion is as follows:

(5) $$\left\{\begin{array}{l} y\geq y_{\min}+\left(j-1\right)d\\ y\leq y_{\min}+jd \end{array}\right.,j=1,2,\ldots ,n.$$

For drones to successfully cover the operation region in the *j*-th subregion, the route of the drones coincided with the center line of this subregion, which can be represented as

(6) $$y_{j}=\frac{y_{\min}+(j-1)d+y_{\min}+jd}{2}=y_{\min}+(j-0.5)d,$$

where $y_{j}$ is the *Y*-coordinate value of the route in the *j*-th subregion.

According to assumption 1, the starting point of the route in the *j*-th subregion can be set as $Q_{start\_j}$, while the ending point can be set as $Q_{end\_j}$. To ensure the uniformity of the pesticide in the operation region, it is necessary to make sure that each point in the operation region has the same sprayed time according to assumption 3. On this premise, $Q_{start\_j}$ and $Q_{end\_j}$ should be as close as possible to the operation region inside the subregion to improve the operation efficiency. However, the covering square at points $Q_{start\_j}$ and $Q_{end\_j}$ cannot overlap the operation region; as a result, the two points $Q_{start\_j}$ and $Q_{end\_j}$ can be set as the point whose covering square just enters the operation region and the point whose covering square just leaves the operation region, respectively.

The ordinate values of points $Q_{start\_j}$ and $Q_{end\_j}$ are given by Equation (6). Then the *X*-coordinate values of points $Q_{start\_j}$ and $Q_{end\_j}$ can be solved and analyzed as described below. The vertices of the convex polygon operation region are $P_{1},\;P_{2}\;\ldots \;P_{m}$, and these m vertices are connected in sequence to form m edges of the convex polygon. The equation of the edges of the convex polygon is as follows:

(7) $$\left\{\begin{array}{l} y=k_{i}x+h_{i}\\ x_{\min\_i}<x<x_{\max\_i}\\ y_{\min\_i}<y<y_{\max\_i} \end{array}\right.,i=1,2,\ldots ,m,$$

where $k_{i},\;h_{i}$ are the slope and *Y*-intercept of the *i*-th edge, $x_{\min\_i},\;x_{\max\_i}$ are the minimum and maximum values of the *X*-coordinate, and $y_{\min\_i},y_{\max\_i}$ are the minimum and maximum values of the *Y*-coordinate of the two vertices making up the *i*-th edge. 

According to Equations (5) and (7), the minimum and maximum values of the *X*-coordinate of the operation region in the *j*-th subregion can be obtained. Let the set of maximum value be $S_{\max}$ and the set of minimum value be $S_{\min}$; then, the geometric relationship between each edge of the convex polygon and the *j*-th subregion can be discussed as follows:

(1) The edges satisfying $y_{\max\_i}<y_{\min}+(j-1)d\cup y_{\min\_i}>y_{\min}+jd$ do not pass through the *j*-th subregion and should be discarded.
(2) The edges satisfying $y_{\min}+(j-1)d\leq y_{\max\_i}\leq y_{\min}+jd\cap y_{\min\_i}<y_{\min}+(j-1)d$ pass through the bottom border line of the *j*-th subregion. The result of $\max(\frac{y_{\max\_i}-h_{i}}{k_{i}},\frac{y_{\min}+(j-1)d-h_{i}}{k_{i}})$ should be included in the set $S_{\max}$, and the result of $\min(\frac{y_{\max\_i}-h_{i}}{k_{i}},\frac{y_{\min}+(j-1)d-h_{i}}{k_{i}})$ should be included in the set $S_{\min}$.
(3) The edges satisfying $y_{\max\_i}>y_{\min}+jd\cap y_{\min}+(j-1)d\leq y_{\min\_i}\leq y_{\min}+jd$ pass through the top border line of the *j*-th subregion. The result of $\max(\frac{y_{\min\_i}-h_{i}}{k_{i}},\frac{y_{\min}+jd-h_{i}}{k_{i}})$ should be included in the set $S_{\max}$, and the result of $\min(\frac{y_{\min\_i}-h_{i}}{k_{i}},\frac{y_{\min}+jd-h_{i}}{k_{i}})$ should be included in the set $S_{\min}$.
(4) The edges satisfying $y_{\min}+(j-1)d\leq y_{\min\_i},y_{\max\_i}\leq y_{\min}+jd$ pass through both the bottom and the top border lines of the *j*-th subregion. The result of $\max(\frac{y_{\min}+(j-1)d-h_{i}}{k_{i}},\frac{y_{\min}+jd-h_{i}}{k_{i}})$ should be included in the set $S_{\max}$, and the result of $\min(\frac{y_{\min}+(j-1)d-h_{i}}{k_{i}},\frac{y_{\min}+jd-h_{i}}{k_{i}})$ should be included in the set $S_{\min}$.

After discussing all the edges of convex polygon, we can get the following results:

(8) $$x_{left\_j}=\min\{S_{\min}\},$$

(9) $$x_{right\_j}=\max\{S_{\max}\},$$

where $x_{left\_j},\;x_{right\_j}$ are the minimum and maximum values of the *X*-coordinate of the operation region in the *j*-th subregion.

The covering square at point $Q_{start\_j}$ enters the operation region exactly, and the covering square at point $Q_{end\_j}$ leaves the operation region exactly, where the length of the covering square’s sides is the spray width d. This can be expressed as follows:

(10a) $$x_{start\_j}=x_{left\_j}-\frac{d}{2},\;x_{end\_j}=x_{right\_j}+\frac{d}{2}$$

(10b) $$x_{start\_j}=x_{right\_j}+\frac{d}{2},\;x_{end\_j}=x_{left\_j}-\frac{d}{2}$$

where $x_{start\_j},\;x_{end\_j}$ correspond to the *X*-coordinate value and the *Y*-coordinate value of $Q_{start\_j}$ and $Q_{end\_j}$**,** respectively; Equation (10a) corresponds to the case where the starting point is on the left side of the operation region, and Equation (10b) corresponds to the case where the starting point is on the right side of operation region.

**Figure 3:**
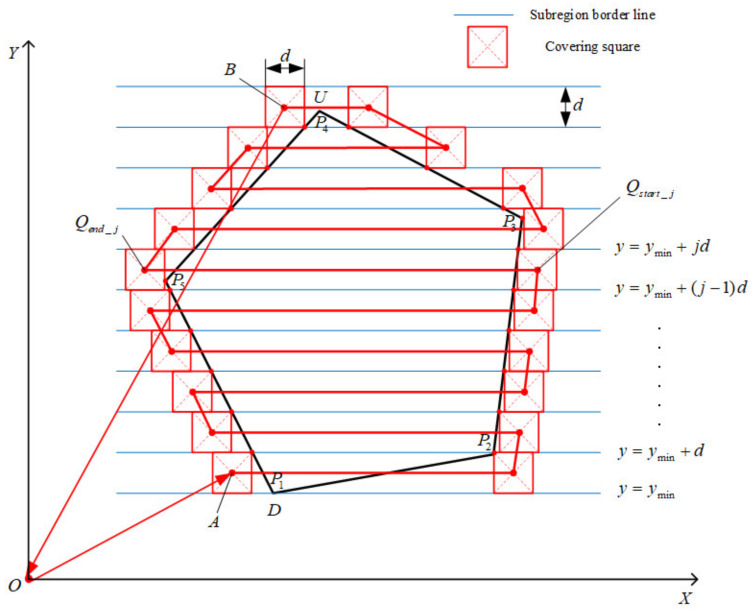
The route generated using the method based on subregion. Point A is the first starting point of the route. Point B is the last ending point of the route. Point O is the takeoff point. Point *D* is the point that has the minimum *Y*-coordinate value among the vertices of the operation region. Point U is the point that has the maximum *Y*-coordinate value among the vertices of the operation region.

Each subregion can be analyzed according to Equations (6)–(10). The starting point can be determined as being on the left or right side of the operation region according to the principle of the reciprocating traversing method. Let the starting point of the route in the first subregion be on the left side of the operation region. Thus, the starting and ending points of the route in all subregions can be obtained. The complete route can be obtained after connecting the starting point and ending point in each subregion, as well as the ending point in the current subregion and the starting point in the next subregion. The whole procedure is shown in Figure 3. 

#### 2.2.2. Routes with Different Initial Heading Angles

The method of route generation based on subregion can only generate one route when the local plane coordinate system is used as the reference to delimit the subregion. To make a comparison, it is necessary to transform the reference coordinate system by rotation to generate different routes. Therefore, the initial heading angle α can be introduced. Let α represent the angle between the flight direction $\vec{n}$ and the positive direction of the *X*-coordinate when the drones start to operate from the first starting point of the route. As shown in Figure 3, the initial heading angle of the generated route in the local plane coordinate system is 0°. 

Let the local plane coordinate system be the coordinate system OXY. The intermediate coordinate system $OX^{\prime }Y^{\prime }$ can be obtained by rotating the coordinate system OXY clockwise around the origin O by an angle of β. In the coordinate system $OX^{\prime }Y^{\prime }$, the coordinates of each vertex in the operation region can be calculated as follows:

(11) $$\left(\begin{array}{cc} x_{i}{}^{\prime } & y_{i}{}^{\prime } \end{array}\right)=\left(\begin{array}{cc} x_{i} & y_{i} \end{array}\right)\left(\begin{array}{cc} \cos\beta & \sin\beta \\ -\sin\beta & \cos\beta \end{array}\right),$$

where $x_{i}{}^{\prime },\;y_{i}{}^{\prime }$ are the *X*-coordinate and the *Y*-coordinate of the *i*-th operation region vertex in the coordinate system $OX^{\prime }Y^{\prime }$, whereas $x_{i},\;y_{i}$ are the *X*-coordinate and the *Y*-coordinate of the operation region vertex in the coordinate system OXY.

**Figure 4:**
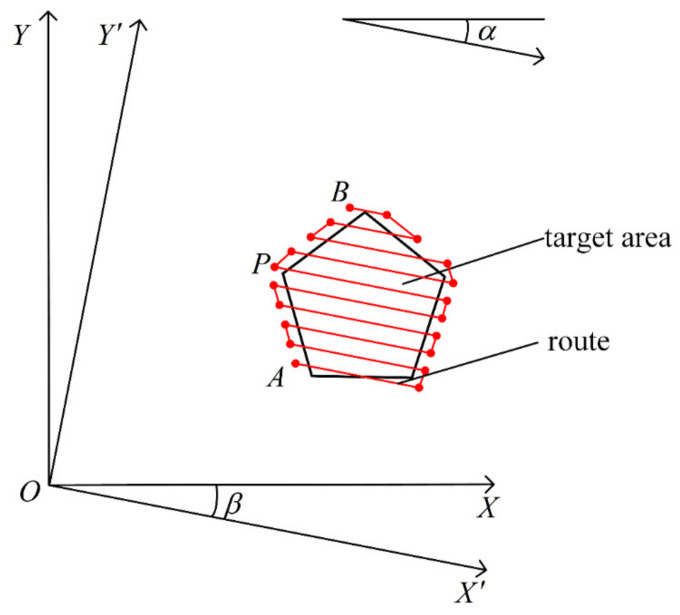
The route with initial heading angle of β. Point A is the first starting point of the route. Point B is the last ending point of the route. Point O is the takeoff point. Point *D* is the point that has the minimum *Y*-coordinate value among the vertices of the operation region.

As shown in Figure 4, in the coordinate system $OX^{\prime }Y^{\prime }$, the starting points and ending points of the route in the n subregions can be obtained according to Equations (3)–(10), which can be transformed back to the coordinate system OXY and connected in sequence to obtain the new route. The transformation formula is as follows:

(12) $$\left(\begin{array}{cc} x_{Q} & y_{Q} \end{array}\right)=\left(\begin{array}{cc} x_{Q}{}^{\prime } & y_{Q}{}^{\prime } \end{array}\right)\left(\begin{array}{cc} \cos\beta & -\sin\beta \\ \sin\beta & \cos\beta \end{array}\right),$$

where $x_{Q},y_{Q}$ are the *X*-coordinate and the *Y*-coordinate of the waypoint in the coordinate system OXY, whereas $x_{Q}{}^{\prime },\;y_{Q}{}^{\prime }$ are the *X*-coordinate and the *Y*-coordinate of the waypoint in the coordinate system $OX^{\prime }Y^{\prime }$.

The initial heading angle of the new generated route is equal to β according to the definition of the initial heading angle, that is to say, α is equal to the angle of rotation from the local plane coordinate system to the intermediate coordinate system. The range of α is from 0–360°. Hence, 360 different routes can be generated according to Equations (3)–(12) in steps of 1°, corresponding to the initial heading angles of 0°,1°,…,359°. 

#### 2.2.3. Evaluation Criteria for Analyzing Different Routes

The different routes with different initial heading angles should be compared to find the optimal one. Before comparison, the evaluation criteria of routes should be provided [28]. Recalling that the goal of route-planning is to improve the operation efficiency as much as possible, there are two approaches: one is to reduce the invalid energy consumption of drones and the other is to avoid the waste of pesticide.

When turning, drones need to reduce the velocity in the current direction and increase the velocity in the direction to be turned. However, the energy consumed by drones in a nonuniform motion is greater than that in a uniform motion [34], indicating that drones need to consume more energy in turning. In addition, [33] theoretically proved that the turning process is less efficient than the rectilinear flight process from the perspectives of energy, distance, and time. Moreover, a greater route distance costs more energy [42]. On the assumption that a drone has a constant pesticide capacity and that these pesticide can be used up in each operation, reducing the waste of pesticide is equivalent to increasing the amount of pesticide applied in the actual operation region, by increasing the coverage area of each operation. Thus, the operation efficiency can be improved. In conclusion, the evaluation criteria for analyzing different routes in this paper were mainly the number of turns, route distance, and pesticide waste rate.

#### 2.2.4. Comparison of Different Routes

According to the evaluation criteria, different routes can be compared. First, the number of turns, route distance, and pesticide waste rate of different routes can be calculated. Then, a control group can be selected to be compared with the different routes.

Analyzing the route corresponding to the initial heading angle α, the number of turns is as follows:

(13) c=2n,

where c is the number of turns, and n is the number of subregions which can be obtained according to Equation (3).

The total route distance can be divided into four parts, namely, the distance from the takeoff point to the first starting point of the route, the distance summation of the routes in each subregion, the distance summation of the route switching subregion, and the distance from the last ending point of the route to the landing point (the same point as the takeoff point). The results can be expressed as follows after analyzing these four parts separately:

(14) $$\left\{\begin{array}{l} l_{1}=\left|\vec{OA}\right|\\ l_{2}=\sum_{j=1}^{n}\left|\vec{Q_{start\_j}Q_{end\_j}}\right|\\ l_{3}=\sum_{j=1}^{n}\left|\vec{Q_{end\_j}Q_{start\_j+1}}\right|\\ l_{4}=\left|\vec{BO}\right| \end{array}\right.,$$

(15) $$l=l_{1}+l_{2}+l_{3}+l_{4},$$

where l is the total route distance, $l_{1}$ is the distance from the takeoff point to the first starting point of the route, $l_{2}$ is the distance summation of the routes in each subregion, $l_{3}$ is the distance summation of the route switching subregion, $l_{4}$ is the distance from the last ending point of the route to the landing point, point O is the takeoff and landing point, point A is the first starting point of the route, point B is the last ending point of the route, point $Q_{start\_j}$ is the starting point of the route in the *j*-th subregion, and point $Q_{end\_j}$ is the ending point of the route in the *j*-th subregion.

The pesticide waste rate is the ratio of the volume of pesticide not used in the operation region to the total volume of pesticide sprayed. Suppose the average velocity of drones during operation is v and the volume of pesticide sprayed per unit time is q; then, the total volume of pesticide sprayed is as follows:

(16) $$V_{total}=\frac{l_{2}}{v}q.$$

The area of the operation region can be obtained using methods provided in [43]. The results can be expressed as follows:

(17) $$S=\frac{\sum_{i=1}^{m-1}\left(x_{i}y_{i+1}-x_{i+1}y_{i}\right)+(x_{m}y_{1}-x_{1}y_{m})}{2},$$

where $x_{i},\;y_{i}$ are the *X*-coordinate value and *Y*-coordinate value of the vertex of the operation region in the local plane coordinate system, and m is the number of vertices of the operation region.

The volume of pesticide sprayed by drones per unit area per unit time can be obtained as

(18) $$a=\frac{q}{d^{2}},$$

where d is the spraying swath width. 

The spraying time at any point in the operation region can be obtained as

(19) $$t=\frac{d}{v}.$$

According to Equations (17)–(19), the volume of pesticide sprayed in the operation region can be obtained as follows:

(20) $$V_{work}=Sat=\frac{Sq}{dv}.$$

According to Equations (16) and (20), the pesticide waste rate can be obtained as

(21) $$\eta =\frac{V_{total}-V_{work}}{V_{total}}=\frac{\frac{l_{2}}{v}q-\frac{Sq}{dv}}{\frac{l_{2}}{v}q}=\frac{l_{2}d-S}{l_{2}d}.$$

Equation (21) shows that the pesticide waste rate η is only related to $l_{2}$, d, and S. Because the spraying swath width and the operation region are input before the route is generated, the value of η changes if and only if the value of $l_{2}$ changes.

After obtaining the number of turns, route distance, and pesticide waste rate of the route corresponding to the initial heading angle of α, 360 routes with different initial heading angles can be compared. When the initial heading angle is not specified for route-planning in the actual operation, the initial heading angle is generally 0°; thus, the route corresponding to the initial heading angle of 0° can be set as the control group. Then, the evaluation function can be determined as follows:

(22) $$f(\alpha )=\varepsilon_{c}\frac{c_{\alpha }}{c_{0}}+\varepsilon_{l}\frac{l_{\alpha }}{l_{0}}+\varepsilon_{\eta }\frac{\eta_{\alpha }}{\eta_{0}},$$

where $c_{\alpha }$ is the number of turns corresponding to the initial heading angle α(0°≤α<360°), $c_{0}$ is the number of turns corresponding to the initial heading angle 0°, $\varepsilon_{c}$ is the weight factor of the number of turns (a larger value denotes a greater role of the number of turns in analyzing different routes), $l_{\alpha }$ is the route distance corresponding to the initial heading angle α, $l_{0}$ is the route distance corresponding to the initial heading angle 0°, $\varepsilon_{l}$ is the weight factor of route distance (a larger value denotes a greater effect of the route distance when analyzing different routes), $\eta_{\alpha }$ is the pesticide waste rate of the route corresponding to the initial heading angle α, $\eta_{0}$ is the pesticide waste rate of the route corresponding to the initial heading angle 0°, and $\varepsilon_{\eta }$ is the weight factor of the pesticide waste rate (a larger value denotes a greater effect of pesticide waste rate when analyzing different routes).

The value of α can be changed to find the optimal route. When α satisfies the minimum value of the evaluation function in Equation (22), the corresponding route is the optimal route, and α at this time is the optimal initial heading angle. 

### 2.3. Coordinate Convertion of Optimal Route

The optimal route obtained using the above procedure is in the local plane coordinate system. Since the drone’s route flight control is applied in the Earth coordinate system, it is important to convert the local plane coordinate system back to the Earth coordinate system. The conversion process can be divided into two parts. First, the local plane coordinate system can be translated back to the UTM projection plane coordinate system using Equation (2). Second, the UTM projection coordinate system can be converted back to the Earth coordinate system through inverse calculation of the UTM projection. The inverse calculation formulas of UTM projection are as follows [37,38]:

(23) $$\left\{\begin{array}{l} \begin{array}{l} \lambda =\lambda_{0}+\frac{1}{\cos\varphi_{f}}\left[\begin{array}{l} D-(1+2\tan^{2}\varphi_{f}-9{e^{\prime }}^{2}\cos^{2}\varphi_{f})\frac{D^{3}}{6}+\\ (5-2{e^{\prime }}^{2}\cos^{2}\varphi_{f}+28\tan^{2}\varphi_{f}-3{e^{\prime }}^{4}\cos^{4}\varphi_{f}-9{e^{\prime }}^{2})\frac{D^{5}}{120} \end{array}\right]\\ \varphi =\varphi_{f}-\frac{N_{f}\tan\varphi_{f}}{R_{f}}\left[\begin{array}{l} \frac{D^{2}}{2}-(5+3\tan^{2}\varphi_{f}+10{e^{\prime }}^{2}\cos^{2}\varphi_{f}-4{e^{\prime }}^{4}\cos^{4}\varphi_{f}-9{e^{\prime }}^{2})\frac{D^{4}}{24}+\\ (61+90\tan^{2}\varphi_{f}+298{e^{\prime }}^{2}\cos^{2}\varphi_{f}+\\ 45\tan^{4}\varphi_{f}-252{e^{\prime }}^{2}-3{e^{\prime }}^{4}\cos^{4}\varphi_{f})\frac{D^{6}}{720} \end{array}\right] \end{array} \end{array}\right.,$$

(24) $$\left\{\begin{array}{l} \varphi_{f}=\frac{y}{K_{\varphi }\alpha }\\ \alpha =\frac{a+b}{2}(1+\frac{n^{2}}{4}+\frac{n^{4}}{16}+\ldots )\\ n=\frac{a-b}{a+b} \end{array}\right.,$$

(25) $$R_{f}=\frac{a(1-e^{2})}{{\left(1-e^{2}\sin^{2}\varphi_{f}\right)}^{\frac{3}{2}}},$$

(26) $$D=\frac{x-FE}{N_{f}K_{\varphi }},$$

where (x, y) are the coordinates of the calculated point in the UTM projection coordinate system, $\varphi_{f}$ is the latitude of the base point, $N_{f}$ is the curvature radius of the prime vertical of the base point, a is the length of the major semi-axis of Earth, b is the length of the minor semi-axis of Earth, e is the first eccentricity of Earth, and the other variables have the same meaning as in Equation (1).

## 3. Results

**Figure 5:**
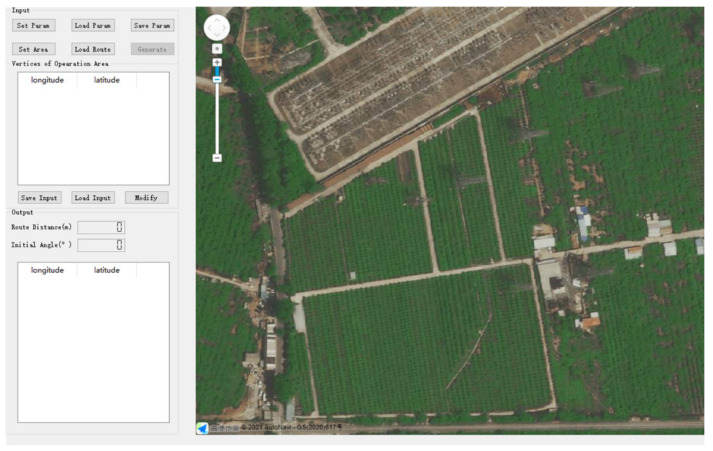
The user interface of the route-planning simulation software. Users can set the operation region by clicking on the map or by sequentially inputting geographical coordinates.

According to the method above, a drone route-planning simulation software was developed using the programming language of C++ and the library of QT. This software embeds the Gaode Map API to display the drone’s real-time position. The software’s user interface (UI) is shown in Figure 5.

The input parameters of simulation are shown in Table 1, where $\varepsilon_{c}=\varepsilon_{l}=\varepsilon_{\eta }=0.333$ indicates that the number of turns, route distance, and pesticide waste rate have the same influence when evaluating the route.

**Figure 6:**
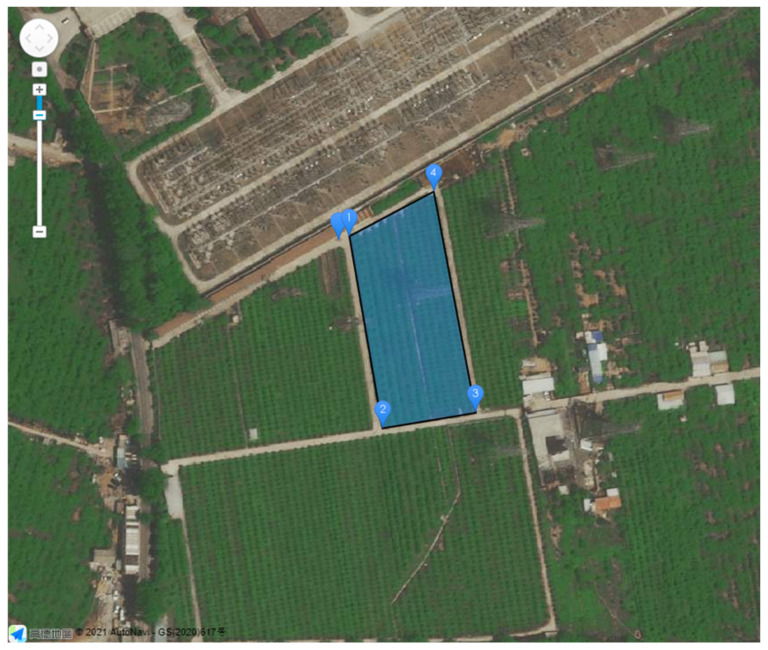
The vertices of the convex polygon operation region and the takeoff point. The unnumbered marker represents the takeoff point, while the numbered markers represent the vertices of the operation region. The blue convex polygon with a black border is the operation region.

The operation region was a certain farmland in Beijing, China with an area of 5167 m^2^. The takeoff point was next to the operation region, as shown in Figure 6. 

**Figure 7:**
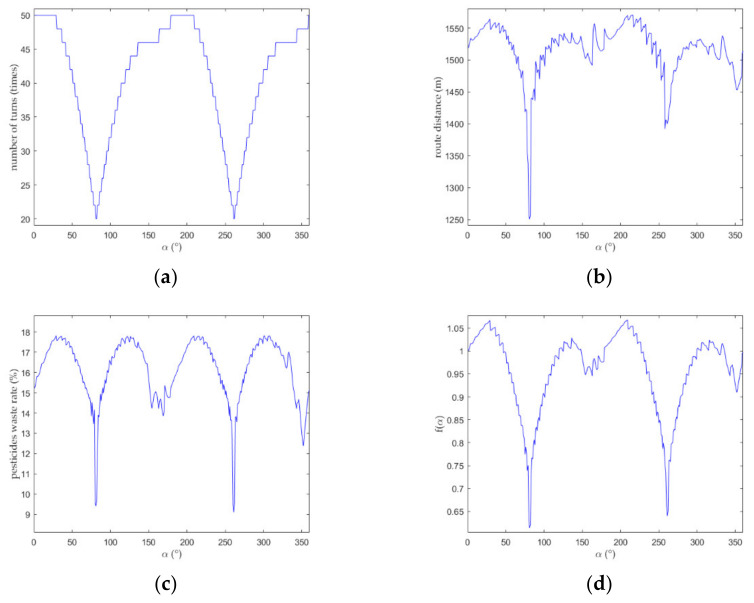
(**a**) Relationship between the number of turns and initial heading angle for different routes; (**b**) relationship between route distance and initial heading angle for different routes; (**c**) relationship between pesticide waste rate and initial heading angle for different routes; (**d**) relationship between evaluation function and initial heading angle for different routes.

**Figure 8:**
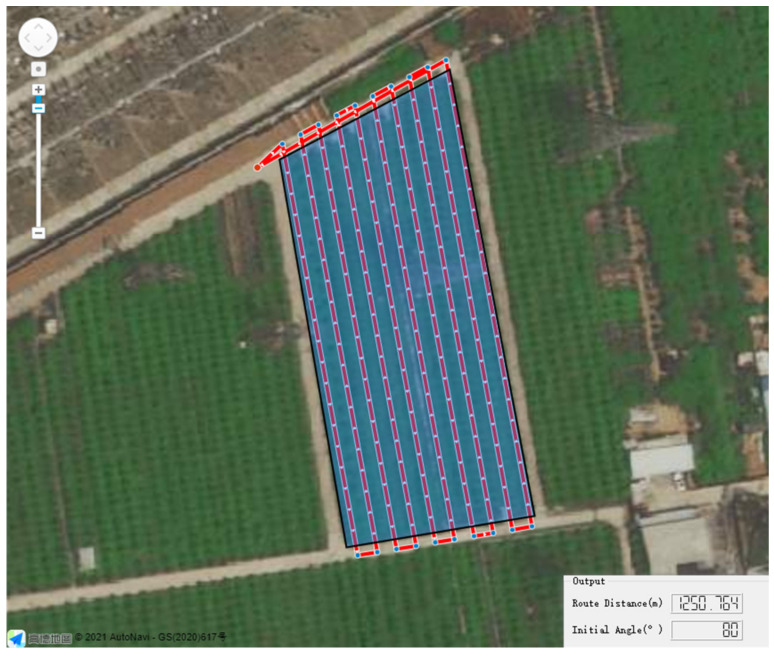
Results of the route-planning simulation software. The red lines with arrows are the calculated optimal route, the blue points are the waypoints, and red point is the takeoff and landing point.

According to Equations (13)–(21), the number of turns, route distance, and pesticide waste rate of the 360 routes corresponding to the initial heading angle of 0°,1°,…,359° were calculated, and the value of the corresponding evaluation function f(α) was calculated according to Equation (22). The result is shown in Figure 7. According to Figure 7a, the minimum value of number of turns corresponded to two values of the initial heading angle, 80° and 260°. As demonstrated in Figure 7b, when the route distance was minimum, the initial heading angle needed to be about 80°. Figure 7c shows that the pesticide waste rate was at a minimum when α=260°. Notably, when α=80°, the pesticide waste rate could reach a value close to minimum. The evaluation function f(α) had a minimum value when α=80° in Figure 7d, suggesting that the best initial heading angle was 80° and the corresponding route was the optimal route. In summary, compared with the control group, i.e., the route with an initial heading angle of 0°, the optimal route was optimized greatly according to all three evaluation criteria and provided the best overall result. The results of the route-planning simulation software are shown in Figure 8. In addition, the whole procedure of generating the optimal route took approximately 1440 ms when using the C++ Chrono Library to count the time.

**Figure 9:**
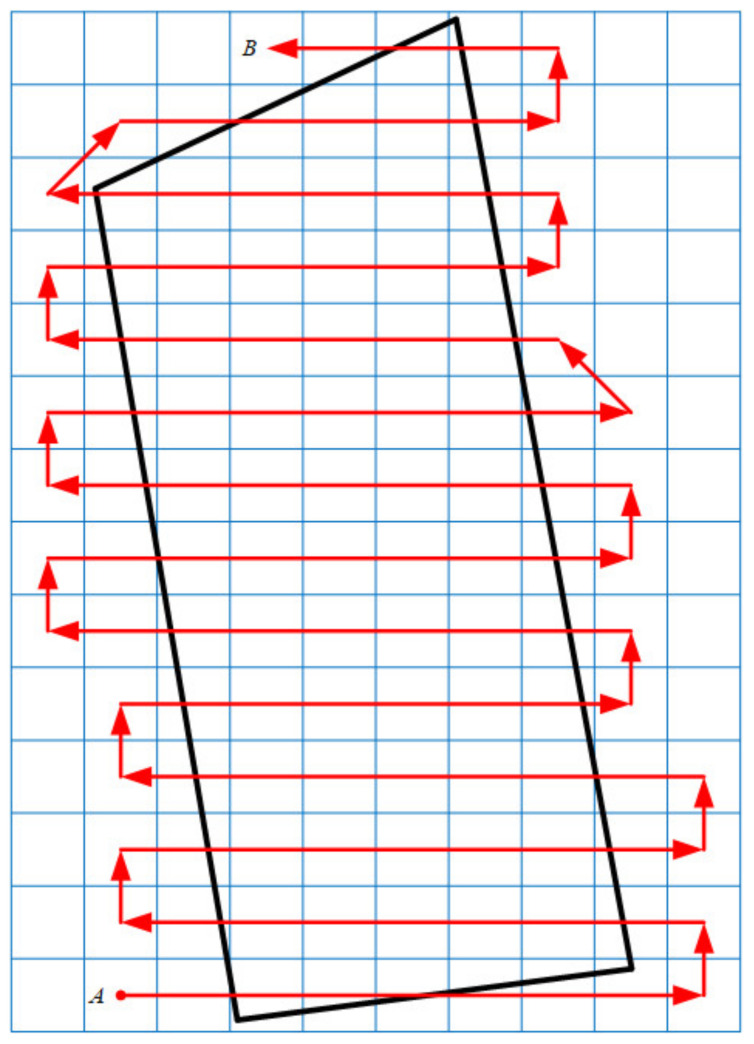
Schematic diagram of the common grid method. The red lines with arrows represent the route. The black polygon represents the operation region. Any grids that overlap with the operation region need to be traversed.

For further comparison, a route with the initial heading angle of 0° was generated using the common grid method mentioned in [18]. The number of turns, route distance, and pesticide waste rate could also be computed using Equations (13)–(21). The principle of the common grid method is shown in Figure 9. Therefore, the route with α=0° generated using the method based on subregion, the route with α=0° generated using the common grid method, and the optimal route (i.e., the route with α=80° generated using the proposed method based on subregion) could be simultaneously compared. The specific results are given in Table 2.

When generating the route with an initial heading angle of 0°, compared with the common grid method, the route generation method based on subregion has the same number of turns, whereas the route distance and pesticide waste rate were reduced by 2.27% and 13.75%, respectively. This means that the method based on subregion achieved the purpose of improving operation efficiency through route generation. When using the route generation method based on subregion, compared with the route with an initial heading angle of 0°, the number of turns, route distance, and pesticide waste rate were reduced by 60%, 17.65%, and 38.18%, respectively, with respect to the route with an initial heading angle of 80°. This means that the optimal route was effectively optimized in all aspects and could greatly improve the operation efficiency. The simulation results fully demonstrate the feasibility of the route-planning algorithm.

## 4. Conclusions and Discussion

In this paper, a route-planning method that generates 360 routes with different initial heading angles and picks the optimal one according to evaluation criteria was developed. The simulation results show that the optimal route performed better considering the number of turns, route distance, and pesticide waste rate. In other words, the optimal route helped improve the operation efficiency by reducing the energy consumption of the drones and the pesticide waste. In conclusion, this method is feasible and suitable for the plant protection of rotor drones. 

However, there were still some limitations in this study. For example, it was considered that the spraying shape of drones was a standard square. However, the spraying shape can be an irregular shape in practice [44]. Furthermore, it was assumed that the spraying operation was uniform at each place, ignoring the influence of wind. When it is windy during operation, many errors might affect the calculation process. During the calculation procedure, the flight velocity is taken as constant regardless of the acceleration and deceleration processes. These influence factors should be considered in the follow-up study. Lastly, this study lacks supporting evidence for the flight experiment; thus, further studies are needed to apply the experimental data and improve the original method.

## Figures and Tables

**Table 1:** Table of input parameters.

Parameter	Value	Parameter	Value
d	5 m	$\varepsilon_{c}$	0.333
q	1 L/min	$\varepsilon_{l}$	0.333
v	5 m/s	$\varepsilon_{\eta }$	0.333

**Table 2:** Comparison of numerical simulation results using three different routes.

Project	Number of Turns (Times)	Route Distance (m)	Pesticide Waste Rate (%)
Subregion α=0°	50	1,518.16	15.2287
Common grid α=0°	50	1,554.20	17.6569
Subregion α=80°	20	1,250.76	9.4148

## Data Availability

Data sharing not applicable.
